# SGLT2 Inhibition After Aortic Valve Replacement: The Role of the Native Valve

**DOI:** 10.1016/j.jacadv.2025.102136

**Published:** 2025-09-18

**Authors:** Antonin Trimaille, Benjamin Marchandot, Olivier Morel

**Affiliations:** a Nouvel Hôpital Civil, Strasbourg University Hospital, Strasbourg, France; b University of Strasbourg, Strasbourg, France; c GERCA, Strasbourg, France; d Hanoi Medical University, Hanoï, Vietnam

We read with great interest the brief report by Abbas et al^1^ on the impact of sodium-glucose cotransporter 2 (SGLT2) inhibitors on bioprosthetic aortic valve dysfunction. In this observational analysis, SGLT2 inhibitor use was associated with a reduced risk of structural valve deterioration. While the study included patients who underwent both surgical and transcatheter aortic valve replacement (TAVR), we believe that the observed effect may be particularly relevant to transcatheter heart valves.

As mentioned by the authors, we recently demonstrated the expression of SGLT2 within stenotic human aortic valves, as well as the activation of the angiotensin system/nicotinamide adenine dinucleotide phosphate oxidase/SGLT2 pro-oxidant pathway in valvular endothelial cells in response to extracellular vesicles entrapped within the native valve.^2^ Notably, SGLT2 inhibitors were shown to prevent these deleterious effects in vitro.

Contrariwise to surgical aortic valve replacement, TAVR leaves the native valve in place, which may continue to negatively interact with the bioprosthesis, particularly through activation of thrombotic pathways that could contribute to long-term structural valve deterioration.^3^ Moreover, the sustained activity of the native valve following TAVR, as evidenced by ^18^F-sodium fluoride positron emission tomography at different time points postprocedure,^4^ may further influence the progression of bioprosthetic valve dysfunction. The DAPA-TAVI (Dapagliflozin in Patients Undergoing Transcatheter Aortic Valve Implantation) trial showed that SGLT2 inhibitors reduced the 1-year composite of all-cause mortality and worsening heart failure compared with standard care in patients undergoing TAVR.^5^ Extended longitudinal follow-up is necessary to determine whether intergroup differences in echocardiographic parameters, particularly transaortic pressure gradients, translate into durable valve performance and incremental clinical benefit from SGLT2 inhibitors. Given the involvement of SGLT2 in the pathological phenotype of diseased aortic valves, it is plausible that the beneficial effects of SGLT2 inhibitors observed in this study may be mediated, at least in part, by mitigating the deleterious role of the native valve. Studies specifically investigating the impact of SGLT2 inhibitors on transcatheter heart valve durability are warranted.
